# Editorial: New advances in cardiac electrophysiology

**DOI:** 10.3389/fcvm.2026.1871300

**Published:** 2026-05-22

**Authors:** Ioanna Koniari, Dimitris Tsiachris

**Affiliations:** 1 Department of Cardiology, General University Hospital of Patras, Pátrai, Greece; 2 First Department of Cardiology, National and Kapodistrian University, “Hippokration” Hospital, Athens, Greece

**Keywords:** bioenvelopes, cardiac devices, catheter ablation, fluoroless, paediatric electrophysiology

Cardiac electrophysiology undergoes a period of continuous transformation, driven by advances in cutting-edge technology, biomaterials, and procedural strategies. The Research Topic “New Advances in Cardiac Electrophysiology” highlights how innovative techniques in catheter ablation and device therapy is reshaping clinical practice. Collectively, the contributions in this special issue emphasize three converging aspects: improving procedural precision, enhancing long-term device outcomes and minimizing patient risk, especially in younger individuals.

A principal development is the emergence of extracellular matrix (ECM)-based bioenvelopes for cardiac implantable electronic devices (CIEDs). Traditional synthetic envelopes have been widely used to mitigate post device implantation complications but are correlated with a foreign-body response that leads to fibrotic encapsulation (1). This process can further lead in the formation of an avascular capsule, which may complicate future procedures and limit tissue vascularization (2). In contrast, ECM-based bioenvelopes—derived from decellularized, non-crosslinked porcine small intestinal submucosa—induce constructive remodeling, angiogenesis, and immunomodulation, promoting the formation of a vascularized and biologically active device pocket.The clinical implications of these biologic ECM based bio envelopes are significant as the need for device revision and replacement is increasing. Fibrotic encapsulation correlated with non-biologic materials can increase procedural complexity during reoperation and further contribute to suboptimal device performance. Observational clinical data suggest that ECM bioenvelopes contribute on improved pocket quality, reduced fibrosis, and easier device mobilization during reoperation, than can be translated into more efficient procedures and better long-term procedural outcomes via improved device-tissue interaction and infection prevention (1). Indeed, device-related infection remains one of the most serious complications of CIED implantation, accompanied with significant morbidity, mortality, and healthcare costs. The incorporation of rifampin and minocycline into biologic envelopes enables localized, sustained antibiotic delivery directly within the pocket, achieving high drug concentrations whereas minimizing systemic exposure. Preclinical data reveal effective bacterial eradication and favorable biocompatibility by combining tissue regeneration with targeted antimicrobial therapy with meaningful benefits over existing non biologic envelopes (1).

In parallel with device therapy advancements, catheter ablation techniques are evolving toward greater precision and safety. A major focus of recent innovation is the reduction of radiation exposure during electrophysiologic procedures. This is particularly relevant in pediatric populations, where cumulative radiation exposure is associated with long-term risks. The integration of three-dimensional electroanatomical mapping systems has enabled fluoroless ablations that maintain procedural efficacy while minimizing radiation.In pediatric atrioventricular nodal reentrant tachycardia (AVNRT), this study demonstrated that nonfluoroscopic radiofrequency ablation can have comparable acute success rates, recurrence outcomes, and safety profiles relative to conventional fluoroscopic techniques (3). Notably, the use of 3D mapping improves anatomical precision, with successful ablation sites more frequently located within the lower triangle of Koch, a region critical for effective and safe slow pathway modification (3). This precise targeting may reduce the atrioventricular node injury, highlighting the additional value of mapping technologies in procedural outcomes' optimization.

The movement toward fluoreless electrophysiological procedures is further exemplified by the development of zero-ray ablation techniques. In the treatment of vasovagal syncope, zero-fluoroscopy cardiac autonomic ganglion ablation using intracardiac ultrasound guidance has revealed promising results in young individuals undergoing high intensity physical training (4). This approach enables precise anatomical localization of ganglionated plexi without fluoroscopical guidance, achieving significant increases in heart rate and symptom burden, with no procedural complications. Such findings underscore the feasibility of fluoroless workflow for autonomic ganglionic plexus ablation and support their adoption in younger populations (4).

Another important study published in this Research Topic demonstrated the expanding role of ventricular arrhythmia catheter ablation in younger children. Historically, concerns regarding procedural risks limited the ablation procedures in very young children (5). However, recent data demonstrates that radiofrequency catheter ablation can be performed with high success and low complication rates even in children under four years of age. With acute success rates approaching 99% in younger children and similar recurrence rates across age groups, these results support that ablation is a viable safe therapeutic option for drug-resistant ventricular arrhythmias in young children. This study also revealed no significant differences in ventricular arrhythmia foci across age groups. Especially, the right ventricular outflow tract was the predominant site in younger and older children, followed by the tricuspid annulus and left septum ventricular arrhythmia foci, respectively. Interestingly, premature ventricular beats combined with ventricular tachycardia more frequently in younger individuals. Such insights emphasize the significance of individualized procedural planning and adaptation of reduced fluoroscopic techniques in pediatric electrophysiology (5).

Collectively, the contributions in this Research Topic illustrate a broader shift toward integrated, precision-based electrophysiologic care. Innovations in biomaterials reduce adverse outcomes post device implantation, while advances in mapping and imaging technologies are enhancing procedural accuracy and safety even with limited or even no fluoroscopic guidance. Simultaneously, expanding clinical experience is redefining the boundaries of electrophysiology interventions in children and young populations, enabling safe and effective treatment. Despite these advances, several challenges remain as long-term clinical data are needed to assess the real-world impact of antibiotic-eluting bioenvelopes. Similarly, the adoption of nonfluoroscopic and zero-ray ablation techniques will be dependent on factors such as operator expertise, access to advanced electrophysiology technologies, and cost considerations. Further research is essential to overcome these barriers and ensure that emerging innovations translate into widespread clinical benefit.

In conclusion, the studies presented in “*New Advances in Cardiac Electrophysiology*” reflect continuous progress in the field. By integrating biologic innovation with advanced procedural technologies, cardiac electrophysiology is rendered safer, more effective, and more personalized even in pediatric populations amending current clinical practice.
